# Latent profile analysis of depression in US adults with obstructive sleep apnea hypopnea syndrome

**DOI:** 10.3389/fpsyt.2024.1398669

**Published:** 2024-04-26

**Authors:** Enguang Li, Fangzhu Ai, Chunguang Liang, Qing Chen, Ying Zhao, Kaiyan Xu, Jie Kong

**Affiliations:** Department of Nursing, Jinzhou Medical University, Jinzhou, China

**Keywords:** latent profile analysis, depression, NHANES, obstructive sleep apnea, OSAHS

## Abstract

**Objective:**

This study used latent profile analysis to explore the level of depression among US adults with obstructive sleep apnea hypopnea syndrome (OSAHS) symptoms and to identify different latent categories of depression to gain insight into the characteristic differences between these categories.

**Methods:**

The data of this study were obtained from the National Health and Nutrition Examination Survey (NHANES) database, and the subjects with OSAHS symptoms were aged 18 years and older. The latent profile analysis (LPA) method was used to fit the latent depression categories in subjects with OSAHS symptoms. The chi-square test, rank sum test, and binary logistic regression were used to analyze the influencing factors of depression subgroups in subjects with OSAHS symptoms.

**Results:**

Three latent profiles were identified: low-level (83.7%), moderate-level (14.5%) and high-level (1.8%) depression. The scores of 9 items in the high-level depression group were higher than those in the other two groups. Among them, item 4 “feeling tired or lack of energy” had the highest score in all categories.

**Conclusion:**

Depression in subjects with OSAHS symptoms can be divided into low-level, moderate-level and high-level depression. There are significant differences among different levels of depression in gender, marital status, PIR, BMI, smoking, general health condition, sleep duration and OSAHS symptom severity.

## Introduction

Obstructive sleep apnea hypopnea syndrome (OSAHS) is a chronic sleep-disordered breathing disease. It is characterized by recurrent collapse or obstruction of the upper airway during sleep, resulting in intermittent hypoxia (IH) and hypercapnia (1). This situation greatly increases the risk of multiple psychiatric disorders in subjects with OSAHS symptoms (2). According to the current study, the prevalence of OSAHS in the adult population of the United States ranges from approximately 2% to 14%. It is higher, up to 20%, in individuals over the age of 60 years (3). As of 2019, nearly 1 billion people worldwide are affected by OSAHS, and the prevalence in some countries even exceeds 50%. Of those affected, China has the most significant number, followed by the United States, Brazil and India (4). In addition, subjects with OSAHS symptoms may face a range of severe affective disorders, which may lead to cognitive decline and have the potential to trigger permanent brain damage (3).

Depression is one of the most common affective disorders and a major related cause of the global burden of mental illness (5). It is characterized by mental symptoms such as low mood, loss of interest, difficulty sleeping, pessimism, and low sense of worth (6, 7). Depression is particularly prevalent in subjects with OSAHS symptoms. According to Melanie Harris et al., in a sleep clinic sample, the incidence of depression in subjects with OSAHS symptoms ranges from 21% to 41% (8). In addition, a longitudinal study by Chen Yihua et al. also confirmed the causal relationship between OSAHS and depression. That is, OSAHS may lead to the occurrence of depression (9). The mechanism of OSAHS causing depression may involve the initiation or aggravation of the pathological process of cerebral small vessel disease (C-SVD) and blood-brain barrier (BBB) dysfunction, thus inducing the occurrence of depression (10). The occurrence and development of depression can also affect the mental health of subjects with OSAHS symptoms, which may lead to decreased sleep quality and poor mental state, thus aggravating the symptoms of OSAHS.

Previous studies mainly focused on exploring the influence of different factors on depression in subjects with OSAHS symptoms, including age, gender, OSAHS symptom severity, and other related factors (11–14). However, these studies all used depression as a variable to diagnose the presence of depression or to assess the severity of depression by assessing the total score of the scale or by setting a cut-off value. However, this approach does not fully reflect the reality of the situation. It ignores the intrinsic characteristics of individuals, and there may be specific subgroups that have not yet been identified. To better understand, we need to take a person-centered approach, observe the relationship of relevant variables among participants, and identify subgroups of individuals based on their response patterns to a set of variables (15).

Latent profile analysis (LPA) is a human-centered statistical method used to identify potential, unobserved subgroups or latent profiles in the data. It aims to discover potential, relatively unique groups in the data that show different patterns or characteristics on the observed variables. LPA is often used to study latent types or subgroups in a population to understand the data better and provide personalized intervention or treatment options (16). In healthcare, LPA has been widely used in a variety of studies, one of which includes the field of sleep medicine (17–19). For example, Wan-Ju Cheng et al. analyzed the endotypes of OSAHS symptoms and found three clusters of pathological endotypes in patients with moderate to severe OSAHS, each of which showed different polysomnographic features and clinical symptom characteristics (20). However, to our knowledge, no study has used LPA to investigate depression in subjects with OSAHS symptoms. Therefore, the primary goal of this study was to use the LPA approach for depression subtype identification in subjects with OSAHS symptoms in the United States to address patient heterogeneity. Secondly, the secondary objective was to investigate the influencing factors of different subtypes of depression to gain insight into the characteristics and related factors of depression in subjects with OSAHS symptoms.

## Methods

### Study participants

**Figure 1:**
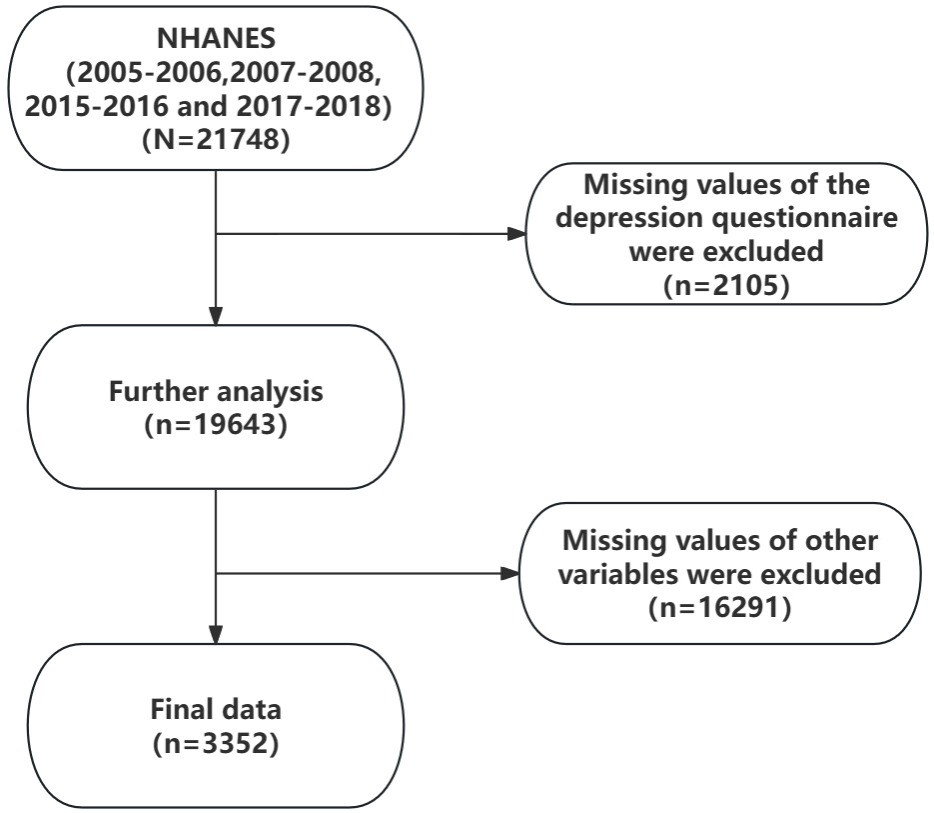
The flowchart of the population screening process. Further analysis: Sample size remaining after excluding missing values from depression questionnaires.

The data used in this study were obtained from the US National Health and Nutrition Examination Survey (NHANES) database. Because this study focused only on subjects with OSAHS symptoms, we chose the sleep questionnaire that included the year in which the question was asked: “How often do you snort/stop breathing?” while years in which the question was not mentioned were excluded. Finally, we selected data from the NHANES database for 2005-2006, 2007-2008, 2015-2016, and 2017-2018 as the sample for this study. This study’s original number of participants was 21748, all aged 18 years and older. After excluding missing values for depression scales, the sample size was reduced to 19643. Subsequently, after excluding missing values of other relevant variables, 3352 subjects were finally included. The sample screening procedure is shown in **Figure 1**. NHANES survey data can be obtained at https://wwwn.cdc.gov/nchs/nhanes/analyticguidelines.aspx.

### Measures

#### Socio-demographic variables

In this study, we used the ratio of family income to poverty (PIR) as the reference standard to measure family income situation. Those with PIR ≤ 1.3 were identified as low-income. However, those with PIR>1.3 were considered a non-low-income population. In addition, we used body mass index (BMI) as a measure of obesity, which was divided into four categories: underweight (BMI, <18.50), normal (18.50 to 24.99), overweight (25.00 to 29.99), and obese (BMI, ≥30).

#### Health behavior variables

Alcohol consumption was categorized into three categories: never, light, and heavy drinking. Never drinking was defined as having a drinking frequency of zero or never drinking in the past 12 months. Light drinking was defined as 1-36 drinks in the previous 12 months, or at least 1-2 drinks per year and up to 2-3 drinks per month. Heavy drinking was defined as drinking more than 37 times in the previous 12 months or drinking at least once a week and up to once a day.

Smoking status was determined based on the subjects’ responses to two questions: “Have you ever smoked more than 100 cigarettes in your lifetime?” and “Do you currently smoke?”. A response of “no more than 100 cigarettes in his lifetime and no current smoker” was defined as never smoking. A response of “smoking more than 100 cigarettes in a lifetime and not currently smoking” was defined as ever smoking. Answering “smoking more than 100 cigarettes in one’s life and now smoking every day, or smoking more than 100 cigarettes in one’s life and now smoking on a few days” was defined as current smoking.

#### Health status variables

The diagnosis of diabetes was based on self-reported judgments of the subject’s responses to the following questions: “Ever been told by a doctor or health professional that you have diabetes or sugar diabetes?”. A response of “yes” was defined as having diabetes, while “no” and “Borderline” were defined as not having diabetes.

General health condition was based on subjects’ responses to “I have some general questions about your health” and “Would you say your health in general is?” The responses to these two questions are defined. Excellent, very good, and good were all defined as good health. Fair was defined as general health status. Poor was defined as poor health.

#### Sleep-related variables

We used seven and nine hours as cut-off points for determining the length of sleep (21, 22). Specifically, sleep duration less than 7 hours was defined as short, sleep duration between 7 and 9 hours was defined as normal, and sleep duration more than 9 hours was defined as long.

OSAHS symptom severity was assessed based on self-report of: “In the past 12 months, how often did you snort, gasp, or stop breathing while you were asleep?”. The selection “Rarely” was recorded as “mild OSAHS symptom,” “Occasionally,” as “moderate OSAHS symptom,” and “Frequently” as “severe OSAHS symptom.”

#### Depression

The Patient Health Questionnaire-9 (PHQ-9) is a commonly used self-rating scale to diagnose depression and assess its severity. Each item on the scale was scored using a Likert four-point scale of 0 (not at all), 1 (a few days), 2 (more than half a day), and 3 (almost every day).The total score ranges from 0 to 27, with higher scores indicating more significant depression. At present, the PHQ-9 has been widely validated in multiple domains, and the results show that the scale has good reliability and validity (23–25). In the present study, the Cronbach alpha coefficient of the PHQ-9, a measure of depression, was 0.858.The assignment of each variable is shown in **Table 1**.

**Table 1:** Independent variable assignment.

Predictive factors	Assignment
**Gender**	“Male” = 1, “Female” = 2
**Age**	Original value entry
**Race**	“Mexican American” = 1, “Other Hispanic” = 2, “Non-Hispanic White” = 3, ”Non-Hispanic Black “ = 4, ”Other Race” = 5
**Education level**	“Less than 9th grade” = 1, “9-11th grade” = 2, “High school graduate” = 3, ”Some college or AA degree “ = 4, ”College graduate or above” = 5
**Marital status**	“Married” = 1, “Widowed” = 2, ”Divorced” = 3, “Separated” = 4, ”Never married” =5, “Living with a partner” = 6
**PIR**	“Low-income(PIR ≤ 1.3)” = 1, “Non-low-income(PIR ≤ 1.3)” = 2
**BMI**	“Underweight(BMI<18.50)” = 0, “Normal weight(18.50~24.99)” = 1, “Overweight(25.00~29.99)” = 2, “Obese(BMI≥30) “ = 3
**Alcohol consumption**	“Never (0)” = 0, “Light(1~36/6~10)” = 1, “Heavy(≥37/1~5)” = 2
**Smoking**	“Never smoked” = 0, “Ever smoked” = 1, “Currently smoked” = 2
**Hypertension**	“Yes” = 1, “No” = 0
**Diabetes**	“Yes” = 1, “No” = 0
**Asthma**	“Yes” = 1, “No” = 0
**Coronary disease**	“Yes” = 1, “No” = 0
**General health condition**	“Good(1~3)” = 1, “Fair(4)” = 2, ”Poor(5)” = 3
**Sleep duration**	“Short(<7 hours)” = 1, “Normal(7~9 hours)” = 2, ”Long(>9 hours)” = 3
**OSAHS symptom severity**	“Mild(1 Rarely)” = 1, “Moderate(2 Occasionally)” = 2, ”Severe(3 Frequently)” = 3

PIR, ratio of family income to poverty; BMI, body mass index; OSAHS, obstructive sleep apnea hypopnea syndrome.

### Statistical analysis

Stata 17.0 (StataCorp LLC, USA) software was used to screen, extract, and combine the NHANES data, and Mplus 8.3 (Muthen and Muthen)and SPSS 25.0 (IBM Corp) software were used for statistical analysis. SPSS 25.0 software was used for statistical description and analysis during data analysis. Quantitative data with normal distribution were expressed as mean ± standard deviation (M ± SD). We used frequency (n) and percentile (%) for representation for qualitative data.

In this study, latent profile analysis of the nine items of the PHQ-9 scale was performed using Mplus 8.3 software. We analyzed latent class by gradually increasing the number of latent classes starting from 1 and simultaneously testing the models’ fit index with different classes. We selected the best-fitting model by comparing the fitting indexes of different models. In selecting the best fitting model, we mainly considered the following model fit indicators: sample corrected aBIC (adjusted BIC, aBIC), Lo-Mendell-Rub test (LMRT) and Entropy were used to evaluate the accuracy of classification. Generally, smaller aBIC values indicate a better model fit (26). We evaluated the classification accuracy using Entropy, which ranges from 0 to 1. When the value of Entropy is closer to 1, the classification is more accurate. In general, when Entropy is greater than or equal to 0.8, the classification accuracy is above 90% (27). BLRT is used to test whether the difference between the K category model and the k-1 category model is significant. When their value is less than 0.05, the K category model has a more substantial improvement in fit compared with the k-1 category model (28). In this study, we will make a comprehensive judgment based on the above fitting indicators to determine the best classification model.

We will use the latent category of depression derived using LPA as the dependent variable when performing univariate analyses. We will use the chi-square test and the Kruskal-Wallis H test for categorical variable comparisons for statistical analysis. For continuous variables that follow a normal distribution, we will use one-way ANOVA for statistical analysis. The variables with statistically significant differences were included in multiple logistic regression analyses to analyze the influencing factors of depression categories in subjects with OSAHS symptoms. A two-sided p-value of less than 0.05 was considered statistically significant.

## Results

### Baseline characteristics

The subjects with OSAHS symptoms in this study were mainly male, accounting for 61.4% of the total sample size. Among them, 3352 subjects with OSAHS symptoms ranged from 20 to 85 years, with an average age of 51.0 (SD = 15.942). The primary characteristics of the participants were mainly Non-Hispanic White (44.7%), married (57.3%), obese (50.1%), never smoking (45.2%), and normal sleep duration (58.1%). Most patients had Some college or AA education degree (31.9%), and most had mild OSAHS symptoms (47.0%). Most of the patients were non-low-income people with good income (72.4%), drank alcohol lightly (68.6%), and self-rated general health condition was good (71.0%). Detailed demographic information of the participants is provided in **Table 2**.

**Table 2:** Baseline information of depressed patients with OSAHS.

Variable	Total number (N=3352)	Variable	Total number (N=3352)
**Gender**		**Alcohol consumption**	
Male	2057 (61.4)	Never	751 (22.4)
Female	1295 (38.6)	Light	2299 (68.6)
**Age**		Heavy	302 (9.0)
	50.66 ± 15.942	**Smoking**	
**Race**		Never smoking	1515 (45.2)
Mexican American	518 (15.5)	Ever smoking	993 (29.6)
Other Hispanic	323 (9.6)	Currently smoking	844 (25.2)
Non-Hispanic White	1497 (44.7)	**Hypertension**	
Non-Hispanic Black	693 (20.7)	Yes	1459 (43.5)
Other Race	321 (9.6)	No	1893 (56.5)
**Education level**		**Diabetes**	
Less than 9th grade	263 (7.8)	Yes	577 (17.2)
9-11th grade	481 (14.3)	No	2775 (82.8)
High school graduate	821 (24.5)	**Asthma**	
Some college or AA degree	1068 (31.9)	Yes	634 (18.9)
College graduate or above	719 (21.4)	No	2718 (81.1)
**Marital status**		**Coronary heart disease**	
Married	1921 (57.3)	Yes	198 (5.9)
Widowed	176 (5.3)	No	3154 (94.1)
Divorced	369 (11.0)	**General health condition**	
Separated	110 (3.3)	Good	2380 (71.0)
Never married	435 (13.0)	General	751 (22.4)
Living with a partner	341 (10.2)	Poor	221 (6.6)
**PIR**		**Sleep duration**	
Low-income	924 (27.6)	Short	1163 (34.7)
Non-low-income	2428 (72.4)	Normal	1948 (58.1)
**BMI**		Long	241 (7.2)
Underweight	39 (1.2)	OSAHS symptom severity	
Normal weight	594 (17.7)	Mild	1575 (47.0)
Overweight	1039 (31.0)	Moderate	964 (28.8)
Obese	1680 (50.1)	Severe	813 (24.3)

PIR, ratio of family income to poverty; BMI, body mass index; OSAHS, obstructive sleep apnea hypopnea syndrome.

### Results of latent profile analysis

In this study, latent profile analysis was performed on the nine items of the PHQ-9 questionnaire, and one to five latent categories were fitted sequentially. The fitting indices of different types of profile models are shown in **Table 3**. The observations showed that Profiles 4 and 5 had P values of LMRT probability greater than 0.05, indicating that they did not reach the significance level and were therefore excluded. At the same time, the aBIC value of Profile 3 is lower than that of Profile 1 and Profile 2, which is more in line with the optimal criteria. Finally, we also need to consider the entropy value. The entropy value of Profile 3 is closer to 1 than Profile 1 and Profile 2, so Profile 3 has the best classification effect. Taking the above analysis together, it can be concluded that Profile 3 is the optimal model.

**Table 3:** Classification of potential fitting models.

Profile	k	Likelihood	aBIC	Entropy	LMRT(*P*)	Proportion
1	18	-34356.574	68802.065			
2	28	-29362.864	58864.045	0.967	0.0000	0.85770/0.14230
3	38	-27374.177	54936.068	0.974	**0.0000**	0.83711/0.14529/0.01760
4	48	-24314.934	48866.981	**0.976**	0.2294	0.80907/0.13634/0.03699/0.01760
5	58	-23637.679	**47561.870**	0.938	0.4858	0.01760/0.05638/0.16766/0.72136/0.03699

aBIC, Adjusted Bayesian Information Criterion; LMRT, Lo-Mendell-Rubin Likelihood Ratio Test.

In order to verify the reliability of the above latent profile analysis results, we calculated the average attribution probability of the three class samples in each class. The results showed that the correct classification probability of the latent class 1 was 99.2%, the latent class 2 was 96.4%, and the latent class 3 was 100.0%. These probabilities are all greater than 90%, indicating that the results of latent profile analysis in this study are relatively reliable. See **Table 4** for details.

**Table 4:** Average Posterior Probabilities for Most Likely Latent Class Membership (Row). by Latent Class (Column).

Class	Profile 1	Profile 2	Profile 3
**Profile 1**	0.992	0.008	0.000
**Profile 2**	0.036	0.964	0.000
**Profile 3**	0.000	0.000	1.000

### Naming of latent profile

**Figure 2:**
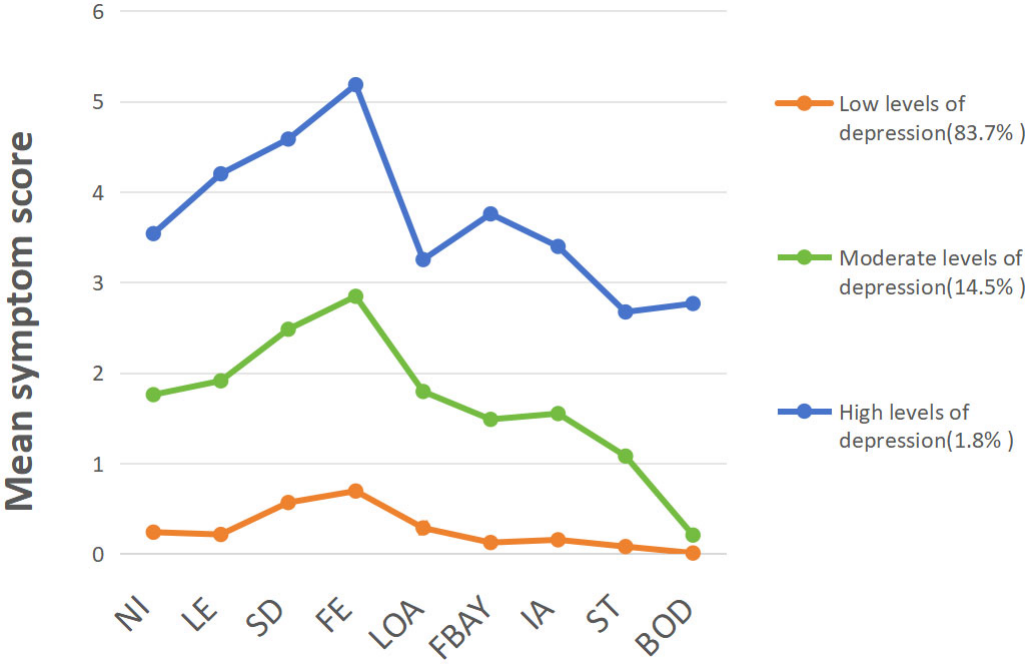
LPA Fit Index for Depression in OSAHS. NI:Have no interest in doing things, LE:Feeling low, depressed, or hopeless, SD: Difficulty falling asleep or sleeping too much, FE:Feeling tired or low in energy, LOA: Poor appetite or overeating, FBAY:Feeling bad about yourself, IA: Difficulty concentrating on things, ST: Moving or speaking slowly or too quickly, BOD: Thought you’d be better off dead.

According to the LPA results, the mean feature scores of each of the nine items in the PHQ-9 are plotted in **Figure 2**. Profile 1 scored significantly lower than Profile 2 and 3 on each item. This group comprised 83.7% of the participants, so we named it “low-level depression” based on its score characteristics. In Profile 2, the score of item 9 was similar to that of Profile 1, and the scores of the remaining eight items were between Profile 1 and Profile 3, accounting for 14.5%. Therefore, we named it “moderate-level depression.” The scores of all items of Profile 3 were significantly higher than those of Profile 1 and Profile 2, and this group was named as having a “high-level depression.”

### Inter-profile characteristic differences

**Table 5** compares differences in demographic characteristics between the three underlying depression types. We used the chi-square test, one-way ANOVA, and Kruskal-Wallis H test to compare the differences in the presence of single risk factors among subjects with OSAHS symptoms with different underlying depression categories. The findings revealed statistically significant differences between patients in different underlying depression categories involving multiple factors. These factors included gender, education level, marital status, PIR, BMI, smoking, hypertension, diabetes, asthma, general health condition, sleep duration, and OSAHS symptom severity. For the remaining categorical differences, we did not observe statistically significant differences. This study considered a two-sided p-value of less than 0.05 statistically significant.

**Table 5:** Demographic characteristics of the different profiles.

Variable	Low-level n = 2806 (83.7%)	Moderate-level n = 487 (14.5%)	High-level n = 59 (1.8%)	χ²/F	*p*
	n (%) or M ± SD	n (%) or M ± SD	n (%) or M ± SD		
**Gender**				71.571	0.000
Male	1810 (64.5)	220 (45.2)	27 (45.8)		
Female	996 (35.5)	267 (54.8)	32 (54.2)		
**Age**				1.026	0.420
	50.96 ± 16.140	49.20 ± 14.677	48.81 ± 15.949		
**Race**				14.042	0.081
Mexican American	457 (16.3)	53 (10.9)	8 (13.6)		
Other Hispanic	270 (9.6)	45 (9.2)	8 (13.6)		
Non-Hispanic White	1235 (44.0)	238 (48.9)	24 (40.7)		
Non-Hispanic Black	569 (20.3)	109 (22.4)	15 (25.4)		
Other Race	275 (9.8)	42 (8.6)	4 (6.8)		
**Education level**				42.885	0.000
Less than 9th grade	208 (7.4)	50 (10.3)	5 (8.5)		
9-11th grade	381 (13.6)	84 (17.2)	16 (27.1)		
High school graduate	671 (23.9)	136 (27.9)	14 (23.7)		
Some college or AA degree	895 (31.9)	157 (32.2)	16 (27.1)		
College graduate or above	651 (23.2)	60 (12.3)	8 (13.6)		
**Marital status**				108.185	0.000
Married	1705 (60.8)	195 (40.0)	21 (35.6)		
Widowed	147 (5.2)	26 (5.3)	3 (5.1)		
Divorced	276 (9.8)	82 (16.8)	11 (18.6)		
Separated	78 (2.8)	30 (6.2)	2 (3.4)		
Never married	321 (11.4)	97 (19.9)	17 (28.8)		
Living with a partner	279 (9.9)	57 (11.7)	5 (8.5)		
**PIR**				139.773	0.000
Low-income	661 (23.6)	238 (48.9)	25 (42.4)		
Non-low-income	2145 (76.4)	249 (51.1)	34 (57.6)		
**BMI**				29.385	0.000
Underweight	29 (1.0)	10 (2.1)	0 (0)		
Normal weight	526 (18.7)	59 (12.1)	9 (15.3)		
Overweight	890 (31.7)	128 (26.3)	21 (35.6)		
Obese	1361 (48.5)	290 (59.5)	29 (49.2)		
**Alcohol consumption**				7.098	0.131
Never	607 (21.6)	130 (26.7)	14 (23.7)		
Light	1948 (69.4)	313 (64.3)	38 (64.4)		
Heavy	251 (8.9)	44 (9.0)	7 (11.9)		
**Smoking**				90.719	0.000
Never smoking	1331 (47.4)	166 (34.1)	18 (30.5)		
Ever smoking	855 (30.5)	126 (25.9)	12 (20.3)		
Currently smoking	620 (22.1)	195 (40.0)	29 (49.2)		
**Hypertension**				24.273	0.000
Yes	1170 (41.7)	261 (53.6)	28 (47.5)		
No	1636 (58.3)	226 (46.4)	31 (52.5)		
**Diabetes**				12.558	0.002
Yes	457 (16.3)	103 (21.1)	17 (28.8)		
No	2349 (83.7)	384 (78.9)	42 (71.2)		
**Asthma**				32.426	0.000
Yes	484 (17.2)	137 (28.1)	13 (22.0)		
No	2322 (82.8)	350 (71.9)	46 (78.0)		
**Coronary heart disease**				3.855	0.146
Yes	157 (5.6)	35 (7.2)	6 (10.2)		
No	2649 (94.4)	452 (92.8)	53 (89.8)		
**General health condition**				344.255	0.000
Good	2155 (76.8)	199 (40.9)	26 (44.1)		
General	541 (19.3)	190 (39.0)	20 (33.9)		
Poor	110 (3.9)	98 (20.1)	13 (22.0)		
**Sleep duration**				60.022	0.000
Short	930 (33.1)	210 (43.1)	23 (39.0)		
Normal	1704 (60.7)	217 (44.6)	27 (45.8)		
Long	172 (6.1)	60 (12.3)	9 (15.3)		
**OSAHS symptom severity**				31.190	0.000
Mild	1367 (48.7)	188 (38.6)	20 (33.9)		
Moderate	796 (28.4)	155 (31.8)	13 (22.0)		
Severe	643 (22.9)	144 (29.6)	26 (44.1)		

PIR, ratio of family income to poverty; BMI, body mass index; OSAHS, obstructive sleep apnea hypopnea syndrome.

In each of the three depression categories, most subjects with OSAHS symptoms were married, obese, and nonlow-income, with good self-reported general health and normal sleep duration. Notably, the proportion of subjects with OSAHS symptoms with low-level depression was higher in men (64.5%) than in women (35.5%). However, the proportion of men with moderate-level and high-level depression decreased (45.2% and 45.8%, respectively). In addition, the smoking rate also increased significantly in people with moderate-level and high-level depression, which were 40.0% and 49.2%, respectively, which were higher than those with low-level depression (22.1%).

### Multiple logistic regression of depression profiles

In this study, the classification of profile characteristics of depression was used as the dependent variable, with reference to high-level and low-level depression, and variables statistically significant in the univariate analysis were studied as independent variables. Subsequently, we included these variables in binary logistic regression models to explore the correlation between observed variables and the classification of each profile. The results showed that PIR in depressive traits did not differ significantly between profiles. Compared with “high level of depression,” we found that the following factors had a significant impact on “low level of depression”: gender, marital status, BMI, smoking, general health condition, sleep duration, and OSAHS symptom severity. Specifically, male (OR= 2.215, P= 0.004), underweight (OR=6.538, P < 0.001), never smoking (OR=2.794, P=0.002) or ever smoking (OR=2.695, P= 0.004), P=0.007), good general health condition(OR=6.605, P<0.001) or general health condition(OR=2.711, P=0.010), normal sleep duration (OR=2.460, P=0.030) and mild to moderate OSAHS symptoms (OR=2.711, P=0.010). p=0.002) (OR=2.338, p=0.016) were more likely to be in a “low-level” depression state. For “moderate-level” depression, BMI and OSAHS symptom severity had a significant impact on it. Specifically, underweight (OR = 5.439, P < 0.001) and mild-to-moderate subjects with OSAHS symptoms (OR = 2.005, P = 0.035) (OR = 2.313, P = 0.021) were more likely to be in the “moderate-level” of depression. Relative to the “low-level depression,” the “moderate-level” was affected by factors such as gender, PIR, BMI, smoking, general health condition, sleep duration, and OSAHS symptom severity. In particular, people with low-income (OR=1.649, P<0.001) were more likely to have a “high-level” of depression. The detailed analysis results of the binary Logistic regression analysis are shown in **Table 6**.

**Table 6:** The results of multiple logistic regression of depression profiles.

Variables	Low VS Moderate		Low VS High		Moderate VS High	
	OR(95%CI)	*p*	OR(95%CI)	*p*	OR(95%CI)	*p*
Gender						
Male	0.475(0.381, 0.593)	0.000	2.215(1.281, 3.830)	0.004	1.053(0.598, 1.854)	0.858
Marital status						
Married	0.737(0.517, 1.050)	0.091	1.063(0.387, 2.925)	0.905	0.784(0.277, 2.214)	0.645
Widowed	0.645(0.371, 1.120)	0.119	1.128(0.254, 5.007)	0.874	0.727(0.156, 3.379)	0.684
Divorced	1.338(0.883, 2.026)	0.170	0.439(0.147, 1.315)	0.141	0.587(0.191, 1.809)	0.354
Separated	1.347(0.767, 2.366)	0.300	0.880(0.162, 4.780)	0.883	1.186(0.214, 6.585)	0.845
Unmarried	1.414(0.946, 2.113)	0.091	0.323(0.115, 0.909)	0.032	0.457(0.158, 1.321)	0.148
PIR						
Low-income	1.649(1.316, 2.065)	0.000	0.866(0.488, 1.537)	0.624	1.428(0.792, 2.577)	0.237
BMI						
Underweight	1.193(0.516, 2.758)	0.680	6.538(2.776, 12.83)	0.000	5.439(1.228,10.553)	0.000
Normal weight	0.591(0.425, 0.821)	0.002	1.133(0.510, 2.517)	0.759	0.670(0.291, 1.538)	0.345
Overweight	0.921(0.717, 1.182)	0.517	0.621(0.342, 1.129)	0.118	0.572(0.308, 1.062)	0.077
Smoking						
Never smoking	0.500(0.385, 0.650)	0.000	2.794(1.479, 5.279)	0.002	1.398(0.723, 2.701)	0.319
Ever smoking	0.578(0.437, 0.766)	0.000	2.695(1.315, 5.524)	0.007	1.558(0.743, 3.266)	0.240
General health condition						
Good	0.157(0.112, 0.220)	0.000	6.605(3.126, 13.95)	0.000	1.036(0.486, 2.210)	0.926
General	0.447(0.317, 0.629)	0.000	2.711(1.273, 5.774)	0.010	1.211(0.567, 2.585)	0.621
Sleep duration						
Short	0.926(0.639, 1.343)	0.686	1.804(0.788, 4.127)	0.163	1.670(0.715, 3.904)	0.236
Normal	0.567(0.393, 0.818)	0.002	2.460(1.094, 5.532)	0.030	1.396(0.606, 3.214)	0.434
OSAHS symptom severity						
Mild	0.739(0.567, 0.964)	0.026	2.711(1.452, 5.064)	0.002	2.005(1.048, 3.834)	0.035
Moderate	0.989(0.749, 1.307)	0.940	2.338(1.169, 4.678)	0.016	2.313(1.133, 4.724)	0.021

PIR, ratio of family income to poverty; BMI, body mass index; OSAHS, obstructive sleep apnea hypopnea syndrome.

## Discussion

In this study, LPA was used to classify depression in subjects with OSAHS symptoms, and three different characteristics were determined according to the scores of each group, namely “low-level depression” (83.7%), “moderate-level depression” (14.5%) and “high-level depression” (1.8%). Among them, 98.2% of subjects with OSAHS symptoms had a moderate or low level of depression.

The three levels of depression showed higher levels in both item 3, “Difficulty falling asleep or sleeping too much”, and item 4 “, Feeling tired or low in energy”. This indicates that depression subjects with OSAHS symptoms generally have the problem of sleeping too long or too short and often feel tired or lack energy. Combined with the results of this study, we conclude that there is A correlation between short sleep duration and depression, which is consistent with the findings of Michael A. Grandner et al (29). In addition, the study by Tiffany J Braley et al. confirmed that sleep disorders, especially OSAHS, may be responsible for the general fatigue felt by patients (30).

The low-level depression group had the lowest and most balanced scores in the other seven items, accounting for 83.7% of the total subjects with OSAHS symptoms. Such patients may have a low probability of depression. However, special attention should be paid to item 3, “Difficulty falling asleep or sleeping too much”, and item 4 “, Feeling tired or low in energy”. This may be because subjects with OSAHS symptoms have upper airway obstruction during sleep, leading to apnea or hypopnea. This disrupts normal sleep cycles and deep sleep, leading to frequent awakenings, which contribute to short sleep duration (31). subjects with OSAHS symptoms may experience decreased blood oxygen levels due to inadequate oxygen supply during apnea or hypopnea. This further affects the body’s energy metabolism and rest recovery process, resulting in patients feeling tired (22). Combined with the results of this study, we believe that early personalized treatment measures should be carried out for people with low-level depression to reduce the severity and symptoms of OSAHS patients and improve their sleep quality and quality of life to prevent them from becoming moderate or high-level depression. Measures include weight control, smoking cessation, adequate sleep duration, and CPAP therapy.

Among subjects with OSAHS symptoms, 14.5% were classified as moderate-level depression. The most prominent item in this group was item 9, “Thought you would be better off dead”, with a score that coincided with low-level depression and was close to zero. This suggests that the risk of suicide is not high for low-level and moderate-level depression in subjects with OSAHS symptoms (32). This may be because depression caused by OSAHS symptoms usually has milder symptoms. Patients are often more likely to accept and respond positively to treatment, which reduces the probability of suicidality (33). The moderate-level depression group is the category most likely to develop into high-level depression, so the level of depression in patients must be detected early and controlled.

1.8% of subjects with OSAHS symptoms were classified as having high-level depression. The scores of item 6, “Feeling bad about yourself”, and item 7 “, Difficulty concentrating on things”, were significantly higher than those of the other two depression categories. This may be because chronic sleep deprivation and poor sleep quality can also hurt mood, leading to a decrease in patients’ self-perception and ability to focus attention (34, 35). A sleep center physician should perform a thorough history and physical examination to determine the presence of major depressive symptoms. Second, they should cooperate with psychiatrists to jointly develop an individualized treatment plan, including cognitive treatments such as pharmacotherapy and cognitive behavioral therapy (36). In addition, patients can also actively conduct self-management, learn to cope with stress and negative emotions and improve self-management skills (37). These measures help to reduce the level of depression as much as possible.

The results of this study showed that gender, marital status, PIR, BMI, smoking, general health condition, sleep duration and OSAHS symptom severity were the influencing factors of depression in OSAHS symptoms patients. Gender plays a crucial role in the development of depression in OSAHS symptoms patients. Due to the influence of biological, hormonal levels and psychosocial factors related to women, the probability of depression in female OSAHS symptoms patients is generally higher. This conclusion is consistent with the results of multiple studies (38–40). In this study, since the proportion of men with OSAHS symptoms is much more significant than that of women, there may be a higher incidence of depression in men than in women, which is also consistent with the results of Min-hwan Lee et al (41). Therefore, sleep physicians should make a comprehensive treatment plan according to the gender characteristics and needs of patients, including sleep therapy, drug treatment and psychological support.

Recent research suggests that a lack of close, confidence-worthy marital relationships may be a vulnerable factor for depression in women living in disadvantaged circumstances (42). The results of the present study also show that unmarried individuals have higher levels of depression than married individuals, which is consistent with the findings of Akihide Inaba (43). Open and effective communication should be maintained between couples, and subjects with OSAHS symptoms can share their feelings and troubles with their spouse to let the other person understand the situation they have experienced (44). In addition, marriage can provide intimacy and companionship and reduce loneliness in patients. Loneliness is often an essential factor in depression, and marriage can provide the emotional connection and support that patients need (42). Therefore, subjects with OSAHS symptoms should be actively involved in sleep therapy as well as other possible treatment methods, such as medication and psychological support.

In this study, we used PIR to indicate household income situation. Income is an essential factor affecting the development of depression in subjects with OSAHS symptoms. This study indicates that people with low income are more likely to suffer from depression than people without low income. This is in line with the findings of Akihide Inaba and Matthew Ridley et al (43, 45). subjects with OSAHS symptoms may need to undergo a range of tests, treatments, and devices, such as sleep monitoring and ventilators. However, the cost of these treatments and devices may be a financial burden for low-income patients. In addition, the level of income may also be associated with the patient’s self-identity and social status. Low income may exacerbate depressive symptoms, leading to feelings of low self-worth and the stress of reduced social status (46). Therefore, it is recommended that low-income groups actively seek appropriate health insurance or social welfare policies to alleviate the financial pressure of treatment and equipment costs.

BMI mainly reflects the weight status of patients. Being overweight or obese is a common risk factor for OSAHS. Excess body weight increases the likelihood of airway obstruction and airway collapse, which leads to an increase in the severity of OSAHS (47). In addition, obesity is associated with chronic inflammation and metabolic disturbances, factors that are thought to be involved in the development of depressive symptoms (48, 49). Therefore, a healthy diet and exercise program is recommended for overweight or obese subjects with OSAHS symptoms with depression in order to lose weight.

In this study, it was found that the order of smoking factors for depression in subjects with OSAHS symptoms was: current smoking greater than former smoking greater than never smoking. This result is consistent with the findings of Tana M. Luger et al (50). Current smokers with OSAHS symptoms may have developed more profound psychological dependence. When faced with stress and anxiety, they were more likely to smoke to relieve their mood. This psychological dependence may increase the risk of depression (50). Based on the above findings, this study suggests that for patients with comorbid smoking, OSAHS and depression, it is necessary to consider various factors to develop corresponding treatment programs and interventions (51). First, smoking cessation interventions are needed, which can involve medication, counselling, or nicotine replacement therapy to help patients quit (52). Physicians also need to conduct a comprehensive physical and psychological assessment of patients to understand the interplay between smoking, OSAHS, and depression and to develop an individualized treatment plan.

General health condition included physical, mental, social, and lifestyle and chronic disease status (53). Sleep disorders and low oxygen supply may lead to physical fatigue and depression in subjects with OSAHS symptoms, which may affect their physiological health. Long-term poor sleep and hypoxia may negatively affect brain function and increase the risk of depression (54). OSAHS itself is a chronic disease, and chronic disease conditions are also associated with depression, so individuals with OSAHS symptoms have a higher risk of depression (55). Therefore, self-rated health is essential to provide family physicians with a practical and straightforward way to identify patients at risk for long-term adverse depressive outcomes and to inform treatment decisions (56). To understand the patient’s overall health, physicians should perform a comprehensive assessment of the patient, including sleep quality, psychological status, social interactions, lifestyle habits, and chronic disease status.

OSAHS can affect the sleep time and sleep quality of patients and further affect the emotional and mental health of patients (57). The results of this study showed A significant correlation between short sleep duration and OSAHS-related depression, which is consistent with the findings of Michael A. Grandner and Amie C Hayley et al (29, 58). In addition, the study by CAROL J. LANG et al. also noted an increased incidence and severity of depression in men with comorbid OSAHS and insomnia (59). This may be because subjects with OSAHS symptoms may wake up frequently or have apnea during the night, affecting their deep sleep and sleep efficiency, so they may need longer sleep to get adequate rest. However, because sleep quality is affected, they may not get enough quality sleep, which may exacerbate depression (22). Therefore, it is recommended that subjects with OSAHS symptoms depression consult a professional physician or sleep specialist for accurate diagnosis and treatment recommendations.

OSAHS symptom severity is an essential factor affecting the risk of depression. In this study, snoring or apnea frequency was used as a subjective indicator to determine the severity of OSAHS, and the results showed a significant correlation between OSAHS symptom severity and depression. This is consistent with the methodology of Sheikh Shoib et al. and further confirms the conclusions of the present study (60). From the perspective of objective accuracy, it is more accurate to use the AHI index as an objective criterion to judge the severity of OSAHS, and several studies have fully confirmed the significant correlation between the AHI index and depression (14, 39). OSAHS symptom severity may affect the sleep quality of patients, and severe OSAHS usually leads to frequent apnea and hypoxemia. This decline in sleep quality may cause symptoms such as mood swings, irritability, fatigue, and even induce or worsen depression. In order to maintain mental health, it is recommended that patients relieve stress and improve their emotional state by communicating with relatives and friends, attending support groups, and seeking professional psychological counselling. If a doctor recommends CPAP devices, they should be used on time every night. CPAP devices may reduce episodes of apnea and hypoxemia by providing airflow of positive pressure to maintain airway patency.

## Limitations

The selection of the subjects with OSAHS symptoms in the NHANES database only relied on patients’ self-reports and did not use professional equipment such as polysomnography (PSG) for diagnosis. This method of data collection may have subjective bias, which in turn affects the objective accuracy of the data. The NHANES database was incomplete, meaning many relevant variables, such as AHI and lowest oxygen saturation, were omitted. Due to the lack of these essential variables, we may not have been able to comprehensively assess their effect on depression in subjects with OSAHS symptoms, which may lead to potential bias.

The effect of menopausal status was not considered in the analysis. Hormonal level changes during menopausal transition may have an impact on the presentation of depression in subjects with OSAHS symptoms. Future studies could take a more comprehensive approach and consider the influence of gender and menopausal status on depression in OSAHS patients to improve the understanding of this complex relationship.

## Conclusion

Depression in subjects with OSAHS symptoms has heterogeneity among individuals, which can be divided into three potential categories, namely low-level depression, moderate-level depression, and high-level depression. There were significant differences in gender, marital status, PIR, BMI, smoking, general health condition, sleep duration, and OSAHS symptom severity among different categories of subjects with OSAHS symptoms. According to the individual characteristics of different categories of subjects with OSAHS symptoms, medical staff can pay special attention to people with low-level depression and provide targeted psychological counseling and support and other intervention programs to reduce their depression levels. Such individualized interventions will be more effective in helping patients cope with the challenges of depression and hopefully improve their overall quality of life.

## Data availability statement

The datasets presented in this study can be found in online repositories. The names of the repository/repositories and accession number(s) can be found in the article/supplementary material.

## Ethics statement

The National Center for Health Statistics (NCHS) Research Ethics Review Board (ERB) reviewed and approved the studies involving human participants. The participants provided their written informed consent to participate in this study.

## Author contributions

EL: Conceptualization, Data curation, Formal analysis, Funding acquisition, Writing – original draft, Writing – review & editing. FA: Investigation, Methodology, Project administration, Writing – original draft, Writing – review & editing. CL: Software, Supervision, Validation, Visualization, Writing – original draft, Writing – review & editing. QC: Project administration, Validation, Writing – original draft, Writing – review & editing. YZ: Resources, Software, Visualization, Writing – original draft, Writing – review & editing. KX: Project administration, Resources, Validation, Visualization, Writing – original draft, Writing – review & editing. JK: Supervision, Validation, Writing – original draft, Writing – review & editing.
